# Modeling of Bimodular Bone Specimen under Four-Point Bending Fatigue Loading

**DOI:** 10.3390/ma15020474

**Published:** 2022-01-08

**Authors:** Yufan Yan, Xianjia Meng, Chuanyong Qu

**Affiliations:** Department of Mechanics, School of Mechanical Engineering, Tianjin University, Tianjin 300350, China; yan_yufan@tju.edu.cn (Y.Y.); xianjiameng@tju.edu.cn (X.M.)

**Keywords:** neutral axis, fatigue, functionally graded beams, bi-modulus, FEM method

## Abstract

The fatigue damage behavior of bone has attracted significant attention in both the mechanical and orthopedic fields. However, due to the complex and hierarchical structure of bone, describing the damage process quantitively or qualitatively is still a significant challenge for researchers in this area. In this study, a nonlinear bi-modulus gradient model was proposed to quantify the neutral axis skewing under fatigue load in a four-point bending test. The digital image correlation technique was used to analyze the tensile and compressive strains during the fatigue process. The results showed that the compressive strain demonstrated an obvious two-stage ascending behavior, whereas the tensile strain revealed a slow upward progression during the fatigue process. Subsequently, a theoretical model was proposed to describe the degradation process of the elastic modulus and the movement of the neutral axis. The changes in the bone properties were determined using the FEM method based on the newly developed model. The results obtained from two different methods exhibited a good degree of consistency. The results obtained in this study are of help in terms of effectively exploring the damage evolution of the bone materials.

## 1. Introduction

**Figure 1:**
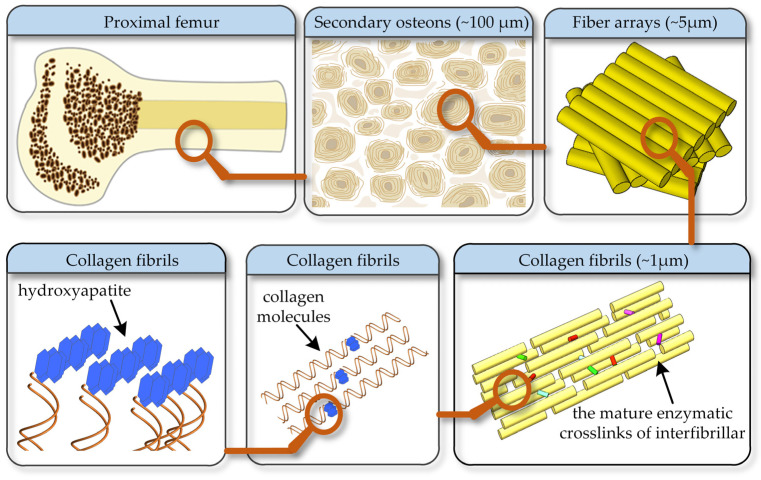
The multistage structure of bone.

Bone, as a supporting organ of our body, is vulnerable to fatigue loadings due to its brittle nature. The damage resistance of bone can be improved by modifying its hierarchical structure. However, it is challenging for researchers in the orthopedic and mechanical fields to model the bone damage and fracture process. Clinically, the bone graft materials are widely used to repair segmental defects and restore mechanical function [1,2,3]. However, the difference in the elastic modulus of these materials and bone tissue can result in a serious ‘‘stress shield’’, which is one of the main causes leading to interface loosening or even implantation failure [1,3]. Therefore, it is vital to study the bone modulus, especially the evolution of the bi-modulus during fatigue damage. As shown in Figure 1, osteons are the main structural unit in the cortical bone, which are assembled from the collagen fiber arrays [4,5]. The fibers are composed of type-I collagen fibers and hydroxyapatites [6]. The type-I collagen fibers, as the organic ductile phase, represent the soft constituent in bones [6]. The hard hydroxyapatite nanocrystals are interspersed with fibers, increasing their stiffness but decreasing their ultimate strain [6,7,8]. The bones exhibit much higher strength and toughness than either of the constituents; thus, these are classified as strong composites with weak constituents [9,10,11,12]. The peculiar hierarchical structures make the bones stiff, strong, tough and light [8]. The bulk mechanical behavior of bones is affected by the contributions of the two basic microconstituents. The collagen fibers exhibit different mechanical behavior in tension and compression, owing to the large aspect ratio and intrinsic properties of the composed molecules [13,14].

It is generally accepted that the brittle hydroxyapatites, which are as fragile as chalk, exhibit different moduli during tensile and compressive loadings [15,16]. The fatigue damage originates from the positions with high local stress in bones, thereby exhibiting different types of cracks as a function of the stress state [17,18]. The diffuse damage occurs under tensile loading, whereas the linear microcracks appear under compressive loading [19,20]. For the same stress in tension or compression, the bones exhibit different tensile and compressive strains, thus signifying the bi-modular effect [13]. The downtrend of the modulus was observed to be more obvious [21], especially at the strain values of 2500 με and 4000 με under tensile and compressive cyclic loading [22]. The tensile strength of bones was observed to be much smaller than their compressive strength under quasi-static loading, whereas the tensile modulus was found to be 6% greater than the compressive modulus [13].

However, during the analysis of the bone fatigue damage, there was no accurate consideration of the bi-modular effects, especially with respect to the stress state during tension and compression. To simplify the experimental analysis, the bones were always considered to be a uniformly linear elastic material without considering their bimodular behavior. As bone is a bimodular material, the neutral axis is not located at the mid-depth of the bone beam [13]. Owing to this behavior, the modulus determined from the classical linear elastic model is neither a tensile nor a compressive modulus. In fact, research studies on the bi-modulus phenomenon have existed for a long time. Many scholars have studied the elastic modulus of tension and compression in pure bending beams. In the longitudinal fibers, Bert’s model made a great contribution to the criterion of positive-negative signs of the strains [23]. Ambartsumyan et. al. conducted outstanding work in this field. They established a bilinear mechanical model based on the positive and negative criteria of the principal stress to explore the different elastic moduli under tension and compression [24].

**Figure 2:**
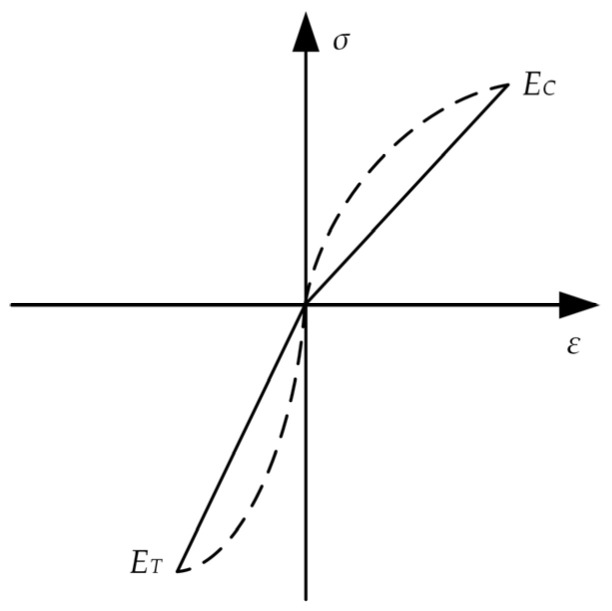
The stress–strain curves depicted by the bi-linear model.

As shown in Figure 2, this model transforms the nonlinear relationship (dashed line) between stress and strain under tensile and compressive loading into the corresponding linear relationship (linear), by making the tangent of the stress-strain curve discontinuous at zero [25]. In the pure bending analysis, both the modulus of the bi-linear model and the normal stress are not continuous at the neutral axis. For bones with an ability to adjust continuously to external loadings, the mechanical properties are more likely to change gradually, thus providing a superior resistance to fatigue. The stress and strain are nonlinear, and the normal stress at the neutral axis is continuous, which is also the focus of interest in this study (Figure 2).

The previously reported mechanical models are largely incapable of explaining the evolution of the modulus as a function of fatigue strain during four-point bending. Additionally, the bimodular functionally graded model of bones has not been provided. For an accurate understanding of the mechanism of bone fatigue damage, it is necessary to take the bimodular effect into consideration, owing to its significant influence on damage resistance.

**Figure 3:**
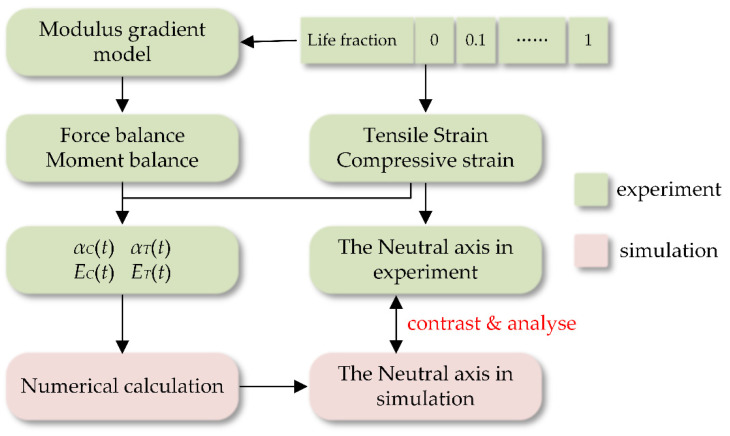
Logic diagram of bone bimodule gradient model.

In this study, the bimodular behavior during cyclic loading is described using a bimodular functionally graded model, based on experimental data. The modulus gradient, under tension and compression, is defined as two logarithmic relationships. The elastic modulus at the neutral axis is assumed to remain constant. Based on the experimental data, the modulus gradients under tension and compression are included, followed by the analysis of the modulus evolution under tension and compression during the cyclic loading. The findings from the finite element analysis are compared with the experimental results to verify the validity of the theoretical model. The argumentation route of the paper is shown in Figure 3. This study enables an effective understanding of the bimodular effects during cyclic loading, which have been largely ignored in the literature due to the complexity of the analysis. The findings obtained herein provide insights into the appropriateness of the bone substitutes for the intended applications, along with helping to develop an inclusive model to explore the complex stress state.

## 2. Materials and Methods

### 2.1. Fatigue Experiment

The tibiae of bovine (approximately 24~36 months old) were collected from a local slaughterhouse in Tianjin, China. The cancellous bone was removed from the ends of the fresh bovine bone using a BOSCH angle grinder (Chengdu, China). Subsequently, a boning knife and hot water were used to clean the remaining tendons and meat scraps in the middle of a compact bone. Thereafter, the bones were soaked in clean water for 24 h. Afterwards, the bones were placed in 1:1 chloroform methanol and 30% H_2_O_2_ for 24 h, respectively, and the cycle was repeated 3 times (24 × 6 h). Later, the bones were soaked in distilled water for 24 h, followed by drying in air. The treated bones were sawed into blocks of dimensions 10 mm × 10 mm × 80 mm along the longitudinal direction. The blocks were ground to the size 6.5 mm × 6.5 mm × 62 mm using silicon carbide (SiC) papers with grit sizes of 400~800. Thereafter, the samples were finely ground to form rectangular specimens (6.0 ± 0.2 mm × 6.0 ± 0.2 mm × 50 ± 0.2 mm) using the SiC papers with grit sizes of 1500~5000. The cooling water was poured continuously throughout the manufacturing process. The long axis of the rectangular specimen was parallel to the longitudinal direction. The angle between each pair of planes was 90°, with a maximum deviation of 2.5%. The height difference was noted to be less than 10 μm, as measured using a 3D surface profiler (ST400, Nanovea, Irvine, CA, USA). Finally, four specimens were used for the pure bending fatigue test. Prior to spraying the speckle and cyclic loading, the specimens were placed in an ultrasonic cleaning setup for 10 min.

**Figure 4:**
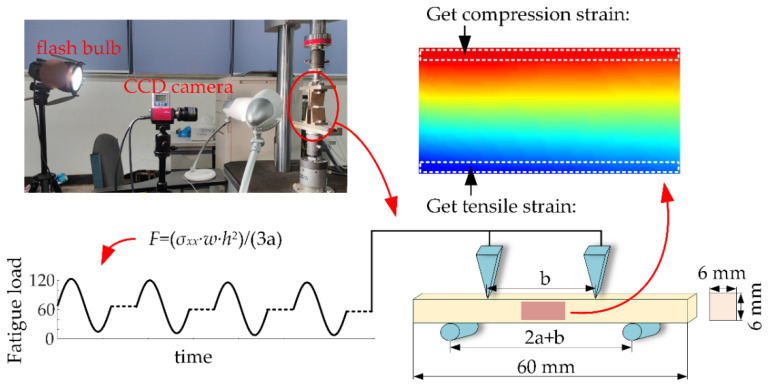
The four-point bending fatigue test.

As shown in Figure 4, a four-point bending test was performed, and the region between the inner contacting points was under the constant bending moment [13,26]. The details of the pure bending test are reported in the literature. The inner span b was 34 mm, whereas the outer span b + 2a was 42 mm, with the parameter a = 4 mm. The sinusoidal waves with a frequency of 10 Hz were generated using ElectroPulsinstron (E10000N, High Wycombe, UK). The load was determined by *F* = (*σ_xx_* × *w* × *h*^2^)/(3a) [13]. a is the distance between the inner and outer supports. *w* is the width of the beam, *h* is the height of the beam, and *σ_xx_* is the applied fatigue stress.

Three samples were loaded for fracturing under quasi-static conditions. The measured ultimate strength of the specimens was 170 ± 8 MPa. The maximum stress *σ_xx_* used in the study was 120 MPa, whereas the minimum stress *σ*_min_ was 12 MPa. The contact radius was 2.0 mm, which was sufficient to avoid the stress concentration. After every 200 fatigue cycles, the loading machine was automatically suspended at 66 MPa stress (Figure 4). The images were swiftly acquired during the off time. All samples were fatigue loaded to attain failure. The measured parameters are shown in Table 1.

### 2.2. Digital Image Correlation

**Figure 5:**
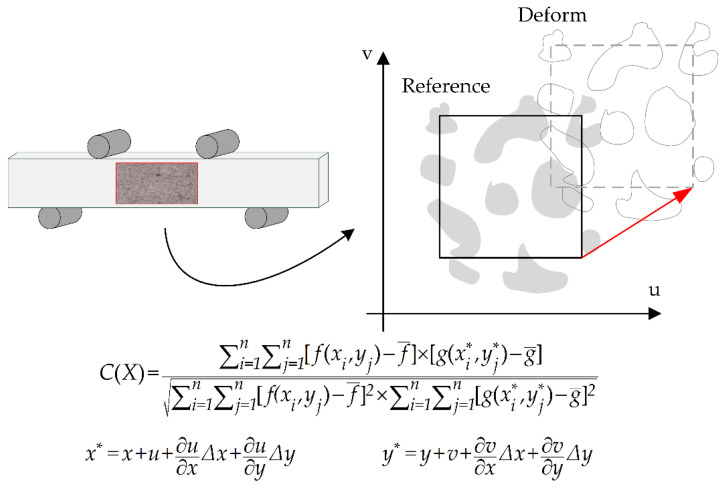
The schematic diagram of the DIC method. For the reference image, *f*(*x*,*y*) is the gray value of the coordinate (*x*,*y*). For the target image, *g*(x*,y*) is the gray level value of the coordinate (x*,y*). $\bar{f}$ and $\bar{g}$ are the average gray values of each image.

The digital image correlation (DIC) technique was used to infer the strain field by comparing the two digital images corresponding to the undeformed and deformed states of the specimen (see Figure 5) [17,27,28]. As a non-contact, real-time and wide-range method, DIC has found widespread use in the field of biomechanics, thus substantiating its use in the current study [29,30].

The image at a stress value of 0 MPa before cyclic loading was used as the reference image. The images at a stress value of 66 MPa at different life fractions were used as the deformed images. After acquiring the images, the displacement fields in the DIC calculation areas were computed by tracing the gray level value of each point in the reference and deformed images as well as performing their image correlation. The correlation coefficient is defined as follows:

### 2.3. The Bimodular Structure of Cortical Bone under Pure Bending Fatigue

**Figure 6:**
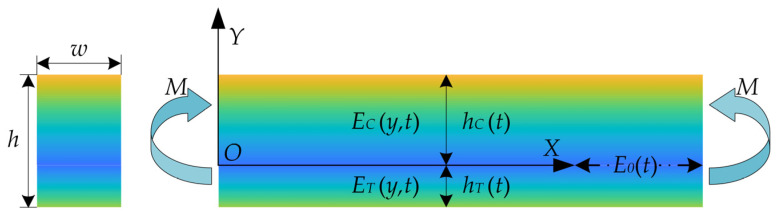
The schematic of a bimodular functionally graded material (FGM) under pure bending.

The cortical bone is a typical bi-modulus nonlinear biomaterial, and its tensile modulus is significantly different from its compression modulus, as verified previously through experiments [13]. The fatigue load can lead to the formation of the microcracks in the cortical bone, and a slight change in the bone structure can be reflected by the corresponding change in the elastic modulus [21]. Based on the four-point bending fatigue load model, a new bi-modulus nonlinear model is presented in this study. As shown in Figure 6, the previously reported theoretical studies on the bi-modulus gradient beams took the elastic modulus $E_{0}\left(t\right)$ at the neutral axis as the boundary [31]. Based on the pure bending model, the elastic modulus under compression is defined as $E_{C}\left(y,t\right)$, whereas the elastic modulus under tension is defined as $E_{T}\left(y,t\right)$. The elastic modulus is closely related to the distance from the neutral axis and the fatigue duration.

At present, the quadratic and exponential functions are generally used for exploring the elastic modulus variation of the functionally gradient beam under pure bending action. On this basis, it was noted that the previously reported models could not describe the changes in the gradient modulus of the bone materials, and the logarithmic function relationship was highly consistent with the actual mechanical properties of bone. Therefore, a gradient model of elastic modulus was expressed in this study in the form of logarithmic function, by considering the dynamic influence of the damage life:

(1) $$E_{C}\left(t\right){=E}_{0}\left(t\right)\left[1-\ln\left(1+\frac{\alpha_{C}\left(t\right)y}{6}\right)\right]{\;0<y<h}_{C}\left(t\right)$$

(2) $$E_{T}\left(t\right){=E}_{0}\left(t\right)\left[1-\ln\left(1+\frac{\alpha_{T}\left(t\right)y}{6}\right)\right]-h_{T}(t)<y<0$$

The parameters $\alpha_{C}\left(t\right)$ and $\alpha_{T}\left(t\right)$ were used to describes the dynamic offset of the neutral axis. In this paper, $\alpha_{C}\left(t\right)$ and $\alpha_{T}\left(t\right)$, which were uncertain at first, were obtained using simultaneous equations based on experimental data. Once the parameters were determined, the elastic modulus gradient model could be determined.

**Figure 7:**
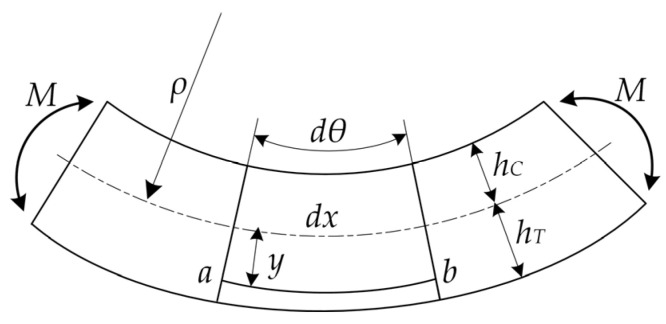
The illustration of displacement under pure bending.

The change in displacement under pure bending is shown in Figure 7. A small section of the pure bending beam is considered. Further, ρ(t) is the distance between the neutral axis and center of curvature. It is known that the tension and compression deformations are absent at the neutral axis. Moreover, ρ(t)dθ is the original length of the pure bent beam, and (ρ(t)+y)dθ is the new length of the micro-beam under the bending load. The strain at the cross-section could be obtained using Equation (3) as:

(3) $$\varepsilon (y,t)=\frac{△l}{l}=\frac{(\rho (t)+y)d\theta -\rho d\theta }{\rho (t)d\theta }=\frac{y}{\rho (t)}$$

A few studies have demonstrated the strain gradient change in the bone materials under pure bending state. In this study, the strain value at the top and bottom of the pure bending beam was extracted. The transverse strain in the cross-section was subsequently transformed as the gradient function. The position of ρ(t) could be obtained from the maximum compressive and tensile strains ($\varepsilon_{C}\left(t\right)$ and $\varepsilon_{T}\left(t\right)$, respectively) of the specimen. As shown in Equation (4), the position of the neutral axis could be determined as:

(4) $$\varepsilon_{C}(t)=\frac{h_{C}\left(t\right)}{\rho (t)}$$

(5) $$\varepsilon_{T}(t)=\frac{h_{T}\left(t\right)}{\rho (t)}$$

(6) $$\varepsilon_{C}{(t)+\varepsilon }_{T}(t)=\frac{h_{C}\left(t\right)}{\rho (t)}+\frac{h_{T}\left(t\right)}{\rho (t)}=\frac{h(t)}{\rho (t)}$$

The transverse strain value in the cross-section of the four-point pure bending beam could be obtained from the experimental data. However, the parameters $\alpha_{C}\left(t\right)$ and $\alpha_{T}\left(t\right)$, related to the fatigue loads, were still unknown. For cases in which the reference values of $\alpha_{C}\left(t\right)$ and $\alpha_{T}\left(t\right)$ can be obtained under different fatigue loads, a constitutive model could have been subsequently developed, which could be used to describe the changes in the bone material modulus gradient.

The transverse compressive and tensile stresses are shown in Equations (7) and (8):

(7) $$\sigma_{C}{(t)=E}_{C}{(t)\varepsilon }_{C}\left(t\right)=E_{C}\left(t\right)y/\rho (t)\;\;\;\;0\leq y\leq h_{C}\left(t\right)$$

(8) $$\sigma_{T}{(t)=E}_{T}{(t)\varepsilon }_{T}\left(t\right)=E_{T}\left(t\right)y/\rho (t)\;-h_{T}\left(t\right)\leq y\leq 0$$

According to the loading condition of the pure bending beam, no external load was present in the transverse direction of the beam; thus, the force balance of the cross-section in a pure bending beam could be obtained using Equation (9) as:

(9) $$\int_{0}^{h_{C}\left(t\right)}\sigma_{C}(t)\mathrm{wdy}+\int_{-h_{T}\left(t\right)}^{0}\sigma_{T}(t)\mathrm{wdy}=0$$

Substituting Equations (7) and (8) into Equation (9) and eliminating $E_{0}\left(t\right)$, ρ(t) and *w*, the following Equation (10) could be obtained:

(10) $$\int_{0}^{h_{C}\left(t\right)}[1-\ln(1-\alpha_{C}(t)y/6)]\mathrm{ydy}+\int_{-h_{T}\left(t\right)}^{0}[1-{\ln(1+\alpha }_{T}(t)y/6)]\mathrm{ydy}=0$$

In order to make the model less cumbersome in the subsequent discussions, $A_{F}^{C}$ and $A_{F}^{T}$ were defined to represent the two integral relations in Equation (10), as shown in Equations (11) and (12). Subsequently, Equation (13), which contains the parameters $\alpha_{C}\left(t\right)$ and $\alpha_{T}\left(t\right)$, could be obtained:

(11) $$\int_{0}^{h_{C}\left(t\right)}[1-\ln(1-\alpha_{C}(t)y/6)]\mathrm{ydy}=A_{F}^{C}{(\alpha }_{C}\left(t)\right)$$

(12) $$\int_{-h_{T}\left(t\right)}^{0}[1-{\ln(1+\alpha }_{T}(t)y/6)]\mathrm{ydy}=A_{F}^{T}{(\alpha }_{T}\left(t)\right)$$

(13) $$A_{F}^{C}{(\alpha }_{C}{(t))+A}_{F}^{T}{(\alpha }_{T}(t))=0$$

The torque balance was similar to the force balance. The pure bending beam was subjected to the force moment *M*, with the force moment equal to *M* in the internal section. Equation (14) could be obtained by factoring *w*, and Equations (15) and (16) could be obtained by substituting Equations (7) and (8) in the two integral relations.

(14) $$\int_{0}^{h_{C}\left(t\right)}\sigma_{C}(t)\mathrm{ydy}+\int_{-h_{T}\left(t\right)}^{0}\sigma_{T}(t)\mathrm{ydy}=M/w$$

(15) $$\int_{0}^{h_{C}\left(t\right)}\sigma_{C}(t)\mathrm{ydy}=E_{0}\left(t)/\rho (t\right)\int_{0}^{h_{C}\left(t\right)}[1-\ln(1-\alpha_{C}{(t)y/6)]y}^{2}\mathrm{dy}$$

(16) $$\int_{-h_{T}\left(t\right)}^{0}\sigma_{T}(t)\mathrm{ydy}=E_{0}\left(t)/\rho (t\right)\int_{-h_{T}\left(t\right)}^{0}[1-{\ln(1+\alpha }_{T}{(t)y/6)]y}^{2}\mathrm{dy}$$

Similarly, the new variables $A_{M}^{C}$ and $A_{M}^{T}$ were defined to replace the complex and lengthy integral relations in Equations (15) and (16), as shown in Equations (17) and (18). On this basis, Equation (19) was obtained from Equations (17) and (18) as:

(17) $$\int_{0}^{h_{C}\left(t\right)}[1-\ln(1-\alpha_{C}\left(t)y/6)\right]y^{2}\mathrm{dy}=A_{M}^{C}{(\alpha }_{C}\left(t)\right)$$

(18) $$\int_{-h_{T}\left(t\right)}^{0}[1-{\ln(1+\alpha }_{T}{(t)y/6)]y}^{2}\mathrm{dy}=A_{M}^{T}{(\alpha }_{T}\left(t)\right)$$

(19) $$A_{M}^{C}{(\alpha }_{C}{(t))+A}_{M}^{T}{(\alpha }_{T}(t))=\frac{M\rho (t)}{{\mathrm{wE}}_{0}\left(t\right)}=\frac{\mathrm{Mh}}{{\mathrm{wE}}_{0}{(t)(\varepsilon }_{C}{(t)+\varepsilon }_{T}\left(t)\right)}$$

From Equations (13) and (19), a set of binary equations containing the unknown parameters $\alpha_{C}\left(t\right)$ and $\alpha_{T}\left(t\right)$ could be obtained. It is worth noting that the two unknown parameters were related to the fatigue duration; thus, their values changed as a function of the fatigue duration. By finding these parameters in the two-element system, the change in the elastic modulus inside the cortical bone could be described.

### 2.4. Finite Element Method Analysis

The MSC. Marc 2015 software was used for the finite element analysis of the bi-modulus bone specimens. The plane stress state was adopted, and the model had a quadrilateral mesh [32]. The mechanical boundary conditions and displacement boundary conditions were consistent with the experiment situation [32]. The material parameters of the finite element model were given according to the gradient modulus distribution function (*E_C_*(*t*) and *E_T_*(*t*)). The neutral layer represented the plane of zero strain formed by the material under external load. The position of the neutral layer was not directly defined in the study. When the gradient modulus function in each fatigue node was determined, the function was substituted into the simulation analysis environment, and then the strain values of the upper and lower sections of the specimen were obtained. The position of the neutral axis from the finite element method was subsequently compared with the experiment to verify the rationality of the model.

## 3. Results

**Figure 8:**
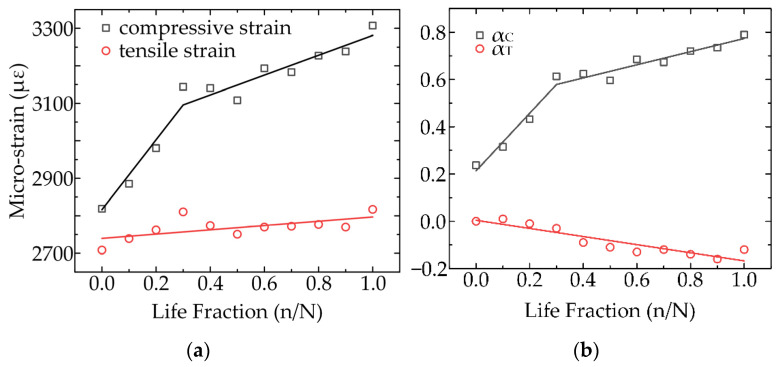
The evolution of the average strain (**a**) and model parameters (**b**) as a function of fatigue.

In the four-point bending test, there was an obvious linear relationship between the transverse strain and the measured position. The position of the neutral axis was closely related to the inconsistency of the tensile and compression strains. Therefore, the position of the neutral axis could be determined from the strain values at the upper and lower sides of the specimen. The strain values of the four specimens were extracted under different fatigue values to determine the position of the neutral axis of the specimens. The results presented in Figure 8a were obtained by averaging the strain values. The strain values at the tensile stage exhibited a linear relationship, with r^2^ = 0.845. The tensile strain on the lower side of the specimen revealed a slight increment of 4% with the loading of the fatigue load. There were significant differences in strain between the upper and lower parts of the specimen. The compressive strain increased rapidly at first until 28% fatigue life was reached. After reaching 28% fatigue life, the strain rose slowly until the specimen broke. The two phases of the compressive strain were noted to be linear with r^2^ = 0.959.

**Figure 9:**
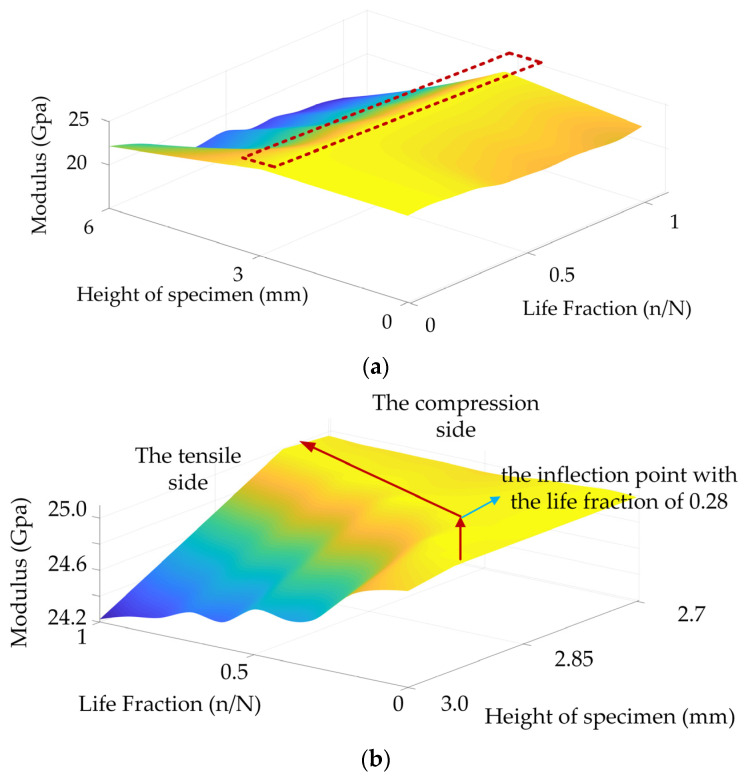
The evolution of modulus during the fatigue process. The change in the modulus gradient inside the pure bending part (**a**). The change in the modulus gradient near the neutral axis (**b**).

The upper and lower strain relationships of the specimens under different degrees of fatigue were obtained experimentally. Eleven groups of strain values were substituted in the two-mode nonlinear model developed in this study to obtain $\alpha_{C}\left(t\right)$ and $\alpha_{T}\left(t\right)$ under different degrees of fatigue, as shown in Figure 8b. $\alpha_{C}\left(t\right)$ exhibited a similar trend to the compression strain on the upper side of the specimen, while $\alpha_{T}\left(t\right)$ exhibited a downward trend. On this basis, the dynamic evolution process of the modulus under fatigue load was obtained, as shown in Figure 9.

As can be observed from Figure 9, the tensile and compression moduli of the specimens decreased to a certain extent. Owing to the uniqueness of the bone materials, the changes in the modulus in different mechanical environments exhibited obvious differences. On increasing the fatigue duration, the neutral axis of the bone material slowly moved towards the tensile point, and the offset of the neutral axis also demonstrated an obvious segmentation. In cases where life fraction was less than 0.28, the dynamic change in the neutral axis was obvious. However, when the life fraction was greater than 0.28, the dynamic migration speed of the neutral axis decreased. It is worth noting that the neutral axis shifted towards compression during the whole fatigue process; however, there was an obvious difference in the offset rate during the offset process.

**Figure 10:**
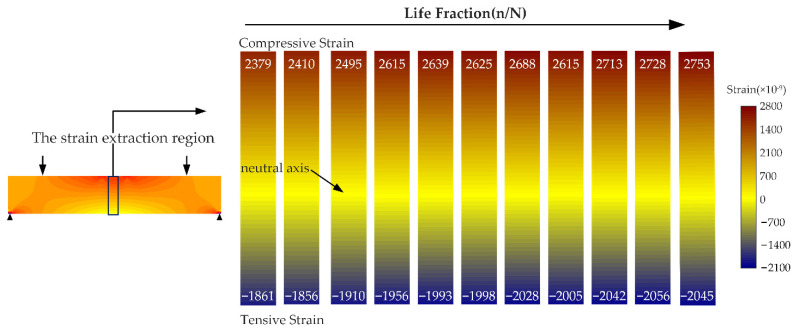
The four-point bending strain distribution.

The position of the neutral axis inside the specimen was determined experimentally. On this basis, the internal parameters $\alpha_{C}\left(t\right)$ and $\alpha_{T}\left(t\right)$ of the two-mode nonlinear model were determined from the force and couple balance. The determined parameters were subsequently introduced in the numerical simulation software Marc to describe the two-mode nonlinear model proposed in this study. In the simulation part, different $\alpha_{C}\left(t\right)$ and $\alpha_{T}\left(t\right)$ values were obtained by solving the equation at each time point. The obtained parameters were used to determine the modulus gradient distribution of the simulation model, and the four-point bending loading process could subsequently be simulated. We extracted the strain values of corresponding regions and imported them into MATLAB 2018b software (see Figure 10).

**Figure 11:**
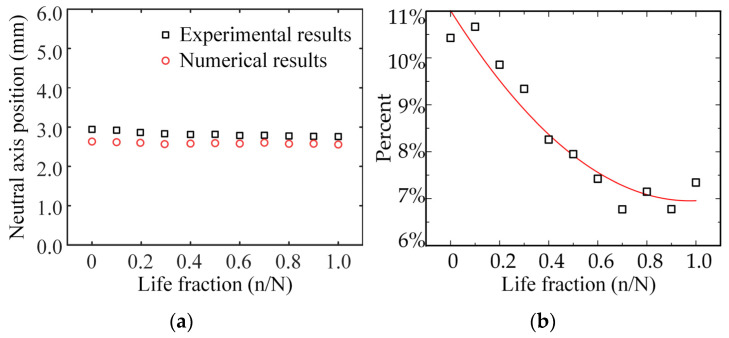
The comparison of experimental and simulation results. The position (**a**) and the deviation (**b**) of the neutral axis.

The experimental results are compared with the simulation results in Figure 11. The simulation results are similar to the experimental findings (see Figure 11a). Upon the increasing of the fatigue degree, the simulation results were observed to become close to the experimental findings (see Figure 11b). The neutral axis positions in the simulation analysis were noted to be lower than the experimental values, and the offset was about 6–11%. Therefore, the nonlinear two-mode gradient model proposed in this paper can reasonably describe the change process of the neutral axis of bone cortex under four-point bending fatigue load.

## 4. Discussion

Due to the complex multi-level structure of bone, the analysis of its mechanical behavior, especially the damage behavior as a function of the stress states, is extremely difficult [14]. The elastic modulus has been widely accepted as a parameter to evaluate the material damage [21]. For a typical brittle material, different tension and compression moduli represent one of the basic mechanical characteristics [13]. Due to the complexity caused by the different tension and compression modulus values, many studies in the literature have ignored this phenomenon in their experiments. Importantly, in order to reflect the actual mechanical state, the use of the gradient damage model with the bi-modulus aspect is vital and meaningful.

The accumulation of damage will affect the deviation of the neutral axis. Specifically, the strain on the compression side increases significantly, while the strain on the tensile side does not change significantly. The neutral axis shifts downward because the strain distribution at the cross section of the specimen changes linearly along the longitudinal axis. Brittle materials can resist large compressive stress, and tensile stress can easily make them fracture. The downward offset of the neutral axis increases the compression portion of the cross section to better resist fatigue loading.

The cortical bone is subjected to diffuse damage on the stretching side [33] and linear microcracks on the compression side [34]. The differences in the damage and the unique characteristics of brittle material lead to different evolutions of the modulus of the compression side and the tensile side in the fatigue process.

The strain distribution of the experiment is similar to the results of previous studies [16]. Some scholars have studied the influence of the fatigue load on the modulus of bone material. It is obvious that the modulus will decrease during fatigue experiments [35,36]. However, previous studies have not discussed the change of the modulus of bone material during fatigue in four-point bending experiments; therefore, this phenomenon was discussed in depth in this paper.

In this article, a nonlinear functionally graded beam method was proposed to describe the offset of the neutral axis and the downtrend of the modulus value. When describing functional gradient characteristics, many functions can be selected according to the actual situation. In theoretical analysis, the exponential function is always selected to describe the functional gradient characteristics of materials [31,37,38]. However, based on the experimental parameters measured in this paper and the actual mechanical properties of bone samples, the logarithmic function was found to be more suitable to describe the functional gradient characteristics of bone fatigue. The stress continuity condition of the neutral layer was introduced because the undetermined constants were increased due to the consideration of different moduli of tension and compression.

During the experiment, the specimens were acquired from the same bone plate. Prior to the fatigue loading, the mechanical properties were analyzed by static loading. The bone specimens with similar strain values were selected to reduce the dispersion.

The theoretical model did not consider the multistage structure and pores of bone, and the four-point bending experiment could not determine the undetermined parameters in the orthotropic model. In order to facilitate the analysis, the research of this paper only considered the elastic modulus along the long axis, and used the Ambartsumyan model to describe the bi-modulus effect. These factors resulted in an error of 6~11% in the neutral axis position of the two groups. The effect of the internal anisotropy of the bone material on the deviation of the neutral axis should be considered in the future research. The findings obtained in this study will help to understand the internal properties of bone materials and provide a benchmark for the future research on bone replacement materials.

## 5. Conclusions

In this study, the weakening of the elastic modulus in the cortical bone was studied both experimentally and theoretically. Through the four-point bending fatigue test, the tensile and compression strain data of the bone specimens were collected, and the position of the neutral axis was subsequently determined. On this basis, a two-mode model in the form of logarithmic function was proposed to describe the mechanical behavior.

Based on the experimental data, the specific values of the two parameters in the model were determined. Afterwards, the results from the finite element analysis were compared with the experimental results to verify the validity of the theoretical model. The downward shift of the neutral axis and gradient weakening of the modulus caused the stress at the tensile point to remain stable, while the stress at the compression point exhibited a downward trend. Thus, the model could effectively reduce the maximum stress in the specimen and enhance the fatigue resistance of the bone material.

## Figures and Tables

**Table 1:** The four-point bending fatigue test and initial strains as a function of the stress state.

Specimen	a (mm)	b (mm)	*ε_C_* (με)	*ε_T_* (με)	*σ_xx_* (MPa)	Ne	N
1	4	34	3157	2953	120	200	8117
2	4	34	2749	2731	120	200	10,962
3	4	34	2726	2604	120	200	9279
4	4	34	2644	2544	120	200	12,665
Average	4	34	2819	2807	120	200	10,270

a = Inner Span; b = Outer Span; *ε_C_* = Compressive Strain; *ε_T_* = Tensile Strain; *σ_xx_* = Fatigue Stress; Ne = Cycle Interval; N = Cycle Number.

## Data Availability

The raw/processed data required to reproduce these findings cannot be shared at this time as the data also form part of an ongoing study. The data presented in this study are available on request from the corresponding author.

## Bone Source Statement

The bovine tibia used in this research was obtained from a slaughtering factory in Tianjin, China. No live subjects were involved in this research.
